# Connecting the public with clinical trial options: The ResearchMatch Trials Today tool

**DOI:** 10.1017/cts.2018.327

**Published:** 2018-11-27

**Authors:** Jill M. Pulley, Rebecca N. Jerome, Gordon R. Bernard, Erik J. Olson, Jason Tan, Consuelo H. Wilkins, Paul A. Harris

**Affiliations:** 1 Vanderbilt Institute for Clinical and Translational Research, Vanderbilt University Medical Center, Nashville, TN, USA; 2 Department of Medicine, Vanderbilt University Medical Center, Nashville, TN, USA; 3 Department of Internal Medicine, Meharry Medical College, Nashville, TN, USA

**Keywords:** Patient information needs, clinical trials, recruitment, trial enrollment, databases

## Abstract

Potential participants seek information about clinical trials for many reasons, but the process can be challenging. We analyzed 101,249 searches in ResearchMatch Trials Today, a free interface to recruiting trials from ClinicalTrials.gov. Searches from March 2015 to November 2016 included a broad range of conditions and healthy volunteer concepts, including 12,649 unique topics. Trials Today data indicate that it is being used to identify trials on a variety of topics.

## Introduction

Members of the public, including patients, seek information about clinical trials for many reasons. Motivations include: exploring trials that might offer different treatment options and learning about interventions under study for a family member’s condition. Yet, there is usually no one within a typical healthcare system with both the expertise and supported time to walk a patient through various trial options. Recent enhancements to ClinicalTrials.gov are intended to further increase public transparency and availability of trial information [1], and some groups have created trial listings or other trial finding assistance [2, 3]. However, the searcher must weigh various search options that are often not optimized for lay audiences. These disconnects can lead to patients missing viable trial options. Thus, trial participants may represent an alarmingly small subset, with many others unfortunately winnowed out by various challenges.

Public desire to find currently recruiting trials dovetails with recruitment needs among investigators [4–6], a well-documented challenge to the efficient translation of new therapies into better treatments for patients [7, 8]. Research has demonstrated that many patients are unaware of how to find information about clinical trials [9, 10] and are often unable to interpret available information [11, 12]. These issues may have even stronger effects in under-represented populations [9].

### Mounting Emphasis on Transparency and Patient Access to Trial Information

Over the last two decades, there have been a number of national activities surrounding the enhancement of public transparency in clinical trials. Expansions to the National Institutes of Health (NIH) ClinicalTrials.gov trial registry have particularly influenced the availability of standard data describing studies in the United States [13, 14].

A driving force in federal activities has been meeting the regulatory requirements [14, 15]. As such, the resulting infrastructure was *not primarily designed to directly support queries by prospective participants.* There is clear need for further development of intermediating tools that help the public to find and interpret trial information.

### Piloting a Potential Solution

We have designed and implemented a novel public-facing interface leveraging data available from clinicaltrials.gov, called ResearchMatch Trials Today, focused particularly on the needs of individuals seeking clinical trial options in the United States. This report describes the use of the tool and lessons learned during early implementation (March 2015–November 2016) after launch to the lay public.

## Methods

### Functionality and Design

Trials Today (https://www.trialstoday.org) uses National Library of Medicine web services [16] to download and process data from ClinicalTrials.gov related to all currently recruiting trials in the United States. Trials Today is updated daily and freely available for use by anyone without registration, with the aim of minimizing potential barriers. It has no advertising and accepts no industry sponsorship. Trials Today resides within ResearchMatch (https://www.researchmatch.org/), a national volunteer registry designed to help “match” volunteers with eligible researchers from a large consortium [17].

The search experience starts with an intuitive interface, where patients and families describe their goals for trial participation (see Table 1), medical condition, and geographic location to identify studies for further exploration. Our team developed framing questions by studying data elements available in each record and focusing on how prospective participants might most easily approach finding the most relevant studies. The tool also includes an advanced option for experienced searchers.

**Table 1:** Patient-selected purpose for Trials Today’s use using the mediated search, ranked by proportion

Topic	No. (%)
I have/had a disease or condition and I am interested in finding studies that test new treatments	29,818 (37.5)
I am not looking for any treatments but I am interested in being a healthy volunteer for research studies that could help other people in the future	17,804 (22.4)
I have/had a disease or condition and I have already tried the currently approved treatment options and they are not working/are no longer working for me	14,471 (18.2)
I am not looking for any treatments but I am interested in studies that figure out ways to diagnose diseases, develop preventative measures, or develop screening processes	8688 (10.9)
I was recently diagnosed with a disease or condition and I am looking for treatment options, even those that are not yet approved	7386 (9.3)
I was recently diagnosed with a disease or condition and there are no available treatments at all	1255 (1.6)
Total	79,422

When a patient begins entering a term (e.g., diabetes), Trials Today maps to concept unique identifiers in the Unified Medical Language System (UMLS—curated by the NIH National Library of Medicine) to gather synonyms for retrieving all relevant trial records and to reduce typographical errors.

During the search, patients see a dynamically generated dashboard displaying the number of studies, sponsors, and conditions meeting their criteria, followed by a list of studies. Studies that are also registered in ResearchMatch are displayed first; results are then prioritized by distance, relevance to the topic, and the trial’s last verification date. The sponsor(s) and condition(s) display adjacent to each study’s title. The full record display organizes information in digestible chunks through tabs guiding participants to specific components (e.g., eligibility, and locations). Inclusion and exclusion criteria are prominent and displayed side-by-side for easier interpretation.

Trials Today also facilitates actionability by offering easy saving and sharing (via creation of a unique URL, rather than requiring an email) to facilitate informed discussion of trial options with providers, friends, and family.

To improve usability, we have made iterative refinements based on valuable feedback from the community member perspective received from the Mid-South Clinical Data Research Network Stakeholder Advisory Council and Vanderbilt’s Effective Health Communication Core.

### Analysis

This report includes data from user searches in Trials Today from its launch in March 2, 2015 through November 30, 2016. Search metrics extracted from server logs have been normalized by only counting unique searches per session.

## Results

From March 2015 through November 2016, the site logged 101,249 searches. Approximately 22% (*n*=21,827) employed the advanced search, while the majority employed the mediated search. Table 1 describes the approach selected by those using the mediated search; the largest proportion of patients were looking for studies testing new treatments for their disease/condition (29.5%). Other subgroups included healthy volunteers (17.6%) and individuals indicating that current treatment options are not effective (14.3%).

Among the 79,542 patients answering whether they are open to early phase research, 89% (*n*=70,777) responded yes. Analysis of queried topics indicated 13,615 different search phrases; after eliminating synonym duplication (e.g., Bassen-Kornzweig disease, and Bassen-Kornzweig syndrome), searches represented 12,649 distinct topics. Of those, 3863 directly mapped to concepts represented in conditions tagged within a trial record, while 8606 did not, likely due to various issues (e.g., misspellings, and extra specificity such as “arthritis in the lower back”). Nonmapped terms were searched as keywords.

A variety of conditions accounted for the 10 most searched for topics in Trials Today (Table 2), including mental health issues (e.g., depression, bipolar disorder), other high prevalence chronic conditions (e.g., obesity, diabetes), and less common diseases with more challenging paths to diagnosis/treatment (e.g., fibromyalgia, idiopathic pulmonary fibrosis).

**Table 2:** Top 10 most frequently searched conditions in Trials Today

Condition	*n*
Depression	2305
Fibromyalgia	1154
Anxiety	1153
Obesity	1031
Peanut allergy	598
Diabetes	581
Attention deficit hyperactivity disorder	518
Bipolar disorder	506
Idiopathic pulmonary fibrosis	479
Any^a^	445

a With regard to searchers who queried the topic “any,” we infer that some users were unaware that they could leave the condition field blank; this potential usability issue will be addressed by the team in improvements to the site’s help materials.

Comparing overlap between the three most popular mediated search questions, results indicated significant concordance across several of the top conditions (Table 3). However, the data also reveal interesting diversity. Individuals interested in new treatments also represented a small unique subset of conditions, including allergy and asthma, while cancer and sleep conditions appeared among the top hits among those interested in diagnosis, prevention, and screening studies.

**Table 3:** Top 10^a^ most frequently searched conditions among three user-selected goals for their Trials Today search

Conditions	Finding trials because treatment not working	Finding trials testing new treatments	Finding trials exploring diagnosis, screening, or prevention
Depression	1	1	1
Fibromyalgia	2	5	9/10^b^
Anxiety	3	4	4
Obesity	4	3	6
Migraine	5	8	
Bipolar disorder	6	10	
Chronic pain	7		
Diabetes	8	7	7
ADHD	9	9	9/10^b^
Multiple sclerosis	10		
Sleep conditions			8
IPF		6	
Allergy		2	
Cancer			3
Dementia or Alzheimer disease			2
Any condition^c^			5

ADHD, attention deficit hyperactivity disorder; IPF, idiopathic pulmonary fibrosis.

a Topics ranked by the relative number of searches per topic in each searcher-selected trial goal category.

b These two conditions had the same number of searches among those exploring diagnosis, screening, or prevention trials.

c With regard to searchers who queried the topic “any,” we infer that some users were unaware that they could leave the condition field blank.

A total of 86,740 individuals indicated gender: 70% (*n*=61,098) were female and 30% (*n*=25,642) were male. Among those specifying age, means ranged from 31 among healthy volunteers to around 40 for other categories. Openness to travel for research also varied; while 6% were only willing to travel 5–10 miles, 63% indicated willingness to travel 25 miles, and others were willing to travel 50 (14%) or even 100 (11.6%) miles. A small but notable 5% were willing to travel 250 miles or more.

## Discussion

Even in its earliest pilot phase, Trials Today logged more than 110,000 searches. The most-searched-for topics likely reflect unmet medical need and emphasize significant motivation among many affected patients to seek new options via trials. The significant majority (almost 90%) open to studies still in a very experimental stage, as well as the more than 30% willing to travel 50 miles or more to a trial site, further speaks to public willingness and desire to participate in research. The diversity among the frequently searched topics also illustrates the attractiveness of trial participation across a range of conditions with particularly challenging paths to effective management, as well as healthy volunteers.

### Lessons Learned and Future Development

While some groups have created trial listing databases or provide hotline-style assistance in identifying possible trial options [2, 3, 18, 19], the burden remains on the patient to select a search path. Future improvements in a number of features related to readability and usability of Trials Today, identified as important opportunities for development in several existing trial search interfaces [20–24], will be focused on further increasing its relevance to individuals interested in identifying trial options.

We are currently building from our pilot Trials Today iteration to include more complex concepts around “high target” inclusion/exclusion criteria. Currently, these criteria in ClinicalTrials.gov are free-text and largely nonstandardized, varying significantly in detail and complexity. In addition, the reading level of these records can be more difficult to read than doctor notes in the electronic health record [25]. Using a data-driven approach to derive a semantic lexicon for eligibility criteria [26], we are developing techniques to assign a distinctive UMLS semantic type for each UMLS-recognizable term, thus creating unambiguous standardized structure from unstructured text. This approach will enhance search precision by including more collection and use of the patient’s demographics, condition, and medications, plus add new filtering based on health history, lifestyle, and other protocol requirements [27].

Our planned incorporation of on-demand presentation of glossary support to users will also improve the readability, a key issue noted across the literature on clinical trial information access by potential participants [20–22]. We anticipate that these enhancements will improve the site’s usability as well as make the tool and its content more accessible by individuals with varying health literacy, an important characteristic with important effects on users’ trial search success and satisfaction [23].

We are also exploring a new feature that will allow searchers to pursue more information about participation in specific studies they identify within Trials Today by having the system broker information exchange and expression of interest to individual research teams. By thus enabling actionability of the information in Trials Today, we hope that interested patients and those assisting them, such as clinicians, patient navigators, and family members, can connect more easily with study staff; we plan to collect metrics regarding user experience with this new feature, including study staff contacts and whether trial enrollment is achieved. Finally, we launched a national awareness campaign focusing on Trials Today in mid-2017, further expanding the reach of this resource.

### Expanding Trials Today’s Use into Additional Applications

From our initial Trials Today tool, we developed and launched a hybridized version for use just at Vanderbilt. This Vanderbilt-branded version of the newly developed Trials Today Local platform returns only Vanderbilt’s recruiting trials and serves as our public-facing trial listing. Further, we have recently begun sharing Trials Today for similar local use at other institutions. This branded localization service is available at medical centers and research networks at no cost (https://projecttrialstoday.org/). Eleven institutions [Montefiore Medical Center/Einstein College of Medicine, We Partner for Research (Georgetown University), Massachusetts General Hospital, University of Arkansas Medical Center College of Medicine, University of Alabama Birmingham, University of Massachusetts, University of Texas Medical Branch, University of Texas Health—San Antonio, University of Texas Health Science Center at Houston, Vanderbilt University, and Washington University in St. Louis] are now using the Trials Today—Local platform as part of their institution’s public trial listing services.

### Limitations

While the volume and variety of searches suggest utility of this tool, the limitations of the current data set preclude quantitative connections with downstream outcomes, such as increases in potential participant contact with study staff, referrals for enrollment, and effects on successful trial recruitment. Collection of such metrics, however, is being incorporated into ongoing enhancements to the site and its features. Further, while we have used lay feedback to improve the interface’s readability of the Trials Today site, ways to optimize Trials Today’s usability are currently being explored. Indeed, opportunities for enhancing usability have been noted by several researchers across a range of trial skearch interfaces [23, 24].

## Conclusions

The ResearchMatch Trials Today tool was created to facilitate identification of trial opportunities for patients across the United States. Usage data to date indicates the resource is being used to identify trials for a range of health conditions, as well as to find opportunities for healthy volunteers to contribute to clinical research. The significant numbers of individuals using Trials Today in early phase work reported here, in the absence of any formal publicity, emphasizes patient need for aid in finding trial options as well as their motivation to pursue study participation. With future planned enhancements, we aim to further empower patients to find clinical trials in which to participate, leading to the discovery of more options and thus greater personal choice for individuals as well as potentially increasing recruitment efficiency in clinical research.
